# Nickel Ion Release in Nickel-Containing Orthodontics Archwires: A Narrative Review of In Vitro and In Vivo Studies

**DOI:** 10.3390/dj13050206

**Published:** 2025-05-06

**Authors:** Angelina Stoyanova-Ivanova, Velizar Georgiev, Jorge N. R. Martins

**Affiliations:** 1 G. Nadjakov Institute of Solid State Physics, Bulgarian Academy of Sciences, 72 Tzarigradsko Chaussee, 1784 Sofia, Bulgaria; angelina@issp.bas.bg (A.S.-I.); velizar@issp.bas.bg (V.G.); 2 Faculdade de Medicina Dentária, Universidade de Lisboa, 1600-277 Lisboa, Portugal; 3 LIBPhys-FCT UID/FIS/04559/2013, 1600-277 Lisboa, Portugal; 4 Grupo de Investigação em Bioquimica e Biologia Oral (GIBBO), Unidade de Investigação em Ciências Orais e Biomédicas (UICOB), 1600-277 Lisboa, Portugal; 5 Centro de Estudos de Medicina Dentária Baseada na Evidência (CEMDBE), 1600-277 Lisboa, Portugal

**Keywords:** artificial saliva, in vitro, in vivo, nickel-containing archwires, nickel release, orthodontic appliances

## Abstract

Nickel-containing orthodontic archwires, particularly those made of nickel-titanium (NiTi) and stainless steel (SS), play a crucial role in orthodontic treatment using the fixed technique due to their mechanical properties. However, concerns regarding nickel-induced allergic reactions, cytotoxicity, and metal ion release, especially nickel-related ones, persist. This narrative review aims to explore recent findings on nickel release from orthodontic appliances, building upon prior systematic reviews by analyzing both in vitro and in vivo studies under various environmental conditions. The databases Web of Science, Scopus, and PubMed were searched for relevant studies that examined the relationship between nickel ion release from nickel-containing archwires and various environmental conditions. The studies found indicate that while metal ion release occurs during short-term treatment, the levels are lower than harmful thresholds, with factors such as pH, corrosion, length of treatment, and environmental influences affecting release rates. Despite this, long-term studies are few and are usually conducted only in an in vitro or in vivo environment, but not both. To establish causal relationships regarding metal ion release, in vivo monitoring of ions like Ni is critical, with further research needed to assess its prolonged effects. Furthermore, collaborative efforts among practitioners, researchers, and regulatory bodies are vital for developing evidence-based guidelines for orthodontic material selection, prioritizing patient safety and addressing metal ion release risks.

## 1. Introduction

Exposure to high nickel concentrations can cause a variety of pathological effects [1]. Fatal cases have been noted following exposure to nickel carbonyl, and by the early 1930s, nickel was recognized as a cause of contact dermatitis. An increase in cases of lung and nasal cancer in workers exposed to nickel was also noted [2,3]. In 2008, nickel was designated “Allergen of the Year” according to Gillette [4]. In their opinion as dermatologists, the frequency of nickel allergy was continuing to grow. These observations prompted greater interest in the impact that nickel has on human health [5]. Nickel is also recognized as a Group 1 carcinogen by the International Agency for Research on Cancer (IARC). However, there is no conclusive evidence linking nickel released from orthodontic appliances to cancer development in patients [6].

The majority of nickel production is used for the manufacture of stainless steel and nickel alloys [7], which have a wide range of applications in the manufacturing of medical equipment, especially orthodontic archwires. These archwires serve as the primary tool for achieving the desired tooth movement and are regarded as the foundation of orthodontic therapy [8]. Although more sophisticated materials and techniques have replaced many traditional procedures, no archwire is flawless or suitable for every stage of therapy [9]. Some of the most commonly used archwires are made from NiTi (nickel-titanium) alloy, with or without additional elements such as copper, as well as from SS (stainless steel).

These wires are some of the most common orthodontic wires used clinically due to their mechanical properties; however, nickel-titanium wires may contain more than 50% nickel, copper-nickel titanium wires typically have less than 50% nickel content, whereas SS wires contain only 8% nickel [10,11,12]. SS wires are easier to work with and less prone to cause allergic reactions than their NiTi counterparts. Because of the 12–13% chromium content in the SS alloy, a thin, passivating layer of chromium oxide can form, preventing corrosion by inhibiting oxygen diffusion into the deeper alloy layers [13]. However, these wires are relatively rigid and possess limited flexibility, which often necessitates more frequent adjustments during treatment [14,15].

On the other hand, NiTi archwires display high elasticity, shape memory, and resistance to permanent deformation. While exposure to high temperatures may result in irreversible deformation, any temporary strain at lower temperatures can be reversed upon reheating [16]. Notwithstanding these benefits, the cytotoxic, allergic, and potentially mutagenic effects linked to nickel content raise concerns about the biocompatibility of NiTi wires [17].

Long-term orthodontic treatment may have detrimental effects on titanium and SS wires due to variations in pH and fluoride concentration. The fact that orthodontic devices corrode is well established; however, little is known about the impact of corrosion on orthodontic treatment and patient health. Research indicates that metal ions are released during orthodontic treatment, but at significantly lower concentrations than those consumed in a typical daily diet. For a deeper understanding, future research in clinically relevant settings is essential [18,19]. Patients undergoing orthodontic treatment may experience allergies for a variety of reasons, including nickel allergies and exposure [11,20]. A study by Zigante et al. [21] supports this, with its results showing that sensitization to nickel and titanium is more common in female subjects.

With the introduction of NiTi alloy in orthodontics, questions arose regarding its biocompatibility, prompting investigations into the matter. One such study was conducted by Wever et al. [22], who performed a comprehensive analysis, combining in vitro and in vivo tests to evaluate the biocompatibility of NiTi alloy. Their results showed that NiTi alloys possess good short-term biological safety due to their low release of ions and high resistance to corrosion. These findings led the authors to conclude that NiTi alloys are biocompatible and safe for clinical applications. Such results are generally found in studies from the last 10 years, which consistently show that the amount of nickel ions released from orthodontic appliances, such as brackets, in patients’ saliva does not rise above a toxic dosage and even decreases after an initial peak [23].

### 1.1. Allergic Reactions to Nickel Released from Nickel-Containing Alloys

The previously mentioned results do not mean that the nickel released from NiTi alloys is free from undesired side effects. Similar to a typical type IV (delayed-type) reaction, allergic reactions in orthodontics are sometimes triggered by immunological hypersensitivity to nickel [24,25]. This reaction occurs in two stages: sensitization, during which immune cells recognize allergens (such as nickel ions) and produce memory T-cells, and elicitation, where repeated exposure triggers the release of inflammatory mediators, such as cytokines. This leads to localized inflammation, which frequently manifests extra-orally as contact dermatitis with signs and symptoms such as swelling, erythema, or, in extreme cases, mouth ulceration. Ions from chromium, cobalt, copper, titanium, and silver can also cause allergic reactions [11]. In addition to the similar to type IV reactions, nickel release from fixed orthodontic appliances can lead to other reactions and manifestations, not only in the oral cavity but also extra-oral at a distance as described in a review by Di Spirito et al. [26]. Over the past few years, research on metal ion release during treatment with orthodontic archwires has increased. A significantly lower concentration of released ions has been observed compared to those consumed in a typical daily diet, but challenges remain in fully understanding the intricate interactions between material characteristics, environmental factors, and patient-specific factors [27,28,29]. In addition, nickel is the most common cause of metal-induced allergic contact dermatitis and elicits more allergic reactions than all other metals combined [30].

### 1.2. Influence of Saliva and Other Environmental Factors on Nickel Release

It is also important to note the role that saliva and other environmental factors play in the release of nickel (Ni) in the oral cavity during orthodontic treatment. Brackets, bands, and archwires in the oral environment are permanently exposed to conditions such as variable (acidic) pH, which can be influenced by dietary intake, temperature, mechanical fatigue, and the susceptibility of alloys to corrosion [31]. Multiple studies [29,32,33] have examined the release of nickel from nickel-containing archwires in a simulated oral environment by immersing these archwires into artificial saliva for different periods of time that correspond to common treatment durations. It has been shown that the level of nickel in saliva and serum increases significantly after the insertion of fixed orthodontic appliances [34]. Despite this, numerous studies have found that the amount of metal ions released by orthodontic appliances is significantly lower than harmful thresholds. The maximum permissible values of these metals in drinking water are significantly higher than the metal ion concentrations reported in saliva. This indicates that, in comparison to other exposures (such as drinking water), the exposure from orthodontic appliances is lower [35]. A contributing factor to this lower exposure is the presence of surface passivation layers, which are composed of oxides of chromium and titanium. These layers help slow ion release by reducing corrosion, but they can deteriorate due to polishing, mechanical wear, or lower pH levels [13]. Another way to further reduce the release of nickel ions is through the addition of coatings on the appliance itself [36,37].

Interestingly, studies [38,39] have linked radiofrequencies from mobile phones to a higher level of nickel ion release in nickel-containing orthodontic archwires. Mortazavi et al. [38] recommend conducting more studies to investigate how radio frequencies from other electronic devices, such as Wi-Fi routers, may affect the release of nickel from these orthodontic archwires. Rajendran et al. [39] also found that using earphones with a mobile phone significantly reduces the phone’s effect on nickel release, further suggesting that close proximity to electronic devices may trigger an increase in ion release.

Systematic literature reviews authored by Mikulewicz and Chojnacka in 2009 and 2010 [31,35] examine in vivo and in vitro studies on the release of metal ions from orthodontic appliances. Both reviews conclude that, overall, the short-term use of orthodontic appliances does not seem to result in the release of toxic levels of nickel and other metal ions. However, information regarding long-term use was lacking at that time and required further study. A meta-analysis of observational studies conducted by Imani et al. in 2019 [40] also concluded that, while studies support the release of small amounts of nickel, which may promote orally induced tolerance in the early stages of treatment, more studies are needed to control factors affecting saliva composition, with larger sample sizes and greater ethnic diversity among patients.

The present review aims to provide an overview of how the understanding of nickel release has evolved and what new information, if any, has been obtained since the aforementioned systematic reviews, to give suggestions for possibilities of future studies and to make recommendations based on the gathered information. It was opted to review the articles below by categorizing the examined studies into two groups, in vitro and in vivo, and analyzing how various factors affect nickel ion release from nickel-containing orthodontic archwires when exposed to different environmental conditions.

## 2. Scope and Sources of Reviewed Literature

The present narrative review aims to provide a comprehensive overview of the existing literature regarding nickel ion release from stainless steel (SS) and nickel-titanium (NiTi) orthodontic archwires. These are among the most prevalent types of archwire alloys used in modern orthodontic practice, and include SS CrNi wires, thermodynamic heat-activated (martensitic active) NiTi and CuNiTi wires, and superelastic (austenitic active) NiTi wires.

To ensure the relevance and breadth of the literature discussed, a focused and purposive search of the available studies was conducted without employing a systematic review methodology. The primary databases searched were Web of Science (WoS), Scopus, and PubMed. The search strategy utilized keywords such as “nickel ion release”, “nickel content dynamics”, “stainless steel orthodontic archwires”, “nickel-titanium archwires”, “in-vivo” and “in-vitro”. The selection criteria for the articles included in this review were as follows: (a) studies had to involve nickel-containing archwires, specifically those made of stainless steel (SS) and nickel-titanium (NiTi) alloys; (b) studies that explored nickel ion release in either in vivo or in vitro conditions were considered; (c) studies investigating the influence of environmental factors, such as pH or other conditions affecting nickel release, were included.

It is important to emphasize that while a structured approach was employed to select the relevant literature, the process did not follow the rigid protocols of systematic reviews. Instead, the approach was designed to offer a narrative synthesis of the current evidence, providing context and critical insight into the factors influencing nickel ion release in orthodontic materials.

**Figure 1:**
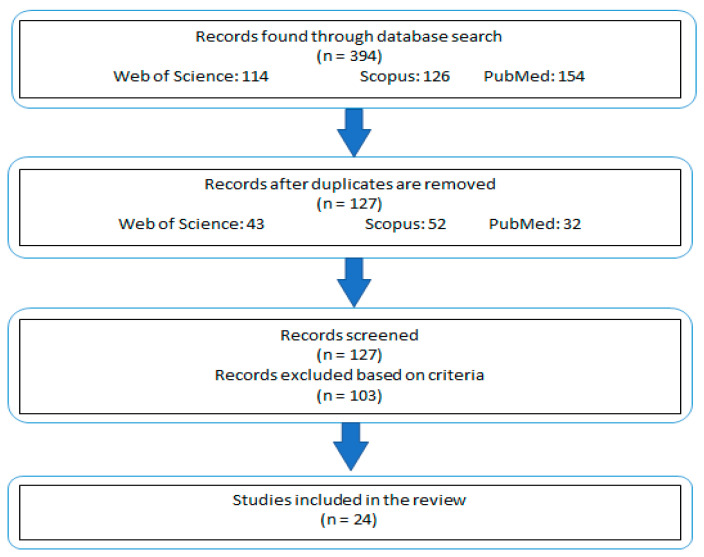
Schematic representation of the study selection and exclusion process.

Studies that did not align with these outlined criteria were excluded from consideration (Figure 1). This approach ensured that the review remained focused on studies most pertinent to the subject matter, while still allowing for a broad and informative exploration of the topic.

## 3. Key Findings from the Literature

For determining the release of ions, atomic absorption spectrometry (AAS) and atomic emission spectroscopy (AES) are the most commonly used analytical methods, as they allow for the measurement of samples with low volumes. Scanning electron microscopy with energy-dispersive spectroscopy (SEM/EDS) is used to determine the surface elemental composition and analyze morphological changes in specific areas of the studied samples.

### 3.1. In Vitro Studies of Ni-Containing Archwires

Most of the in vitro studies reviewed in this work use artificial saliva to simulate the oral environment. Table 1 presents the composition of the artificial saliva used in each reviewed study that employed this immersion medium.

Many in vitro studies focus on how artificial saliva affects ion release from nickel-containing archwires over a set period. For example, Cioffi et al. [41] examined pseudoelastic NiTi archwires under simulated physiological conditions (artificial saliva) to assess the combined effects of strain and fluoridated media. They found no nickel ion release during stress-induced austenite-to-martensite transformation, suggesting that the NiTi surface resists phase transformation under tensile stress. However, prolonged exposure to fluorides significantly increased ion release, prompting the authors to recommend further research on fluoride’s short-term effects.

Following with the topic of fluoride effects, Pastor et al. [42] conducted a study in 2023 which examined some more commonly used archwires after immersing them in different mouthwashes. The results of that study demonstrated that mouthwashes increased the release of nickel, which, as noted by the authors, can cause hypersensitivity in some patients. Thus, they recommend, care should be taken when using mouthwashes during treatment that utilizes orthodontic archwires.

Mirjalili et al. [18] conducted a study with a similar approach, immersing archwires into artificial saliva. They studied localized corrosion and the effects of pre-passivation treatment using potentiodynamic and potentiostatic polarization techniques. Based on their results, the authors concluded that NiTi did not exhibit pitting corrosion in artificial saliva, while stainless steel showed only a marginally beneficial effect. Additionally, they found that an artificial crevice had no effect on corrosion behavior in fluoridated media. Pre-passivation treatment was found to have a positive effect on the pitting corrosion of both alloy types in the presence of fluoride ions.

Also using artificial saliva, Didovic et al. [43] studied NiTi archwires as well as stainless steel (SS) brackets, bands, and ligatures. The findings showed that the fixed appliance’s various components had different surface morphologies due to different production techniques. In their as-received condition, the SS bands and brackets exhibited signs of pitting corrosion. During immersion, adhesive coatings formed on the SS brackets and ligatures; however, protective oxide layers were not observed on any of the components. Additionally, salt precipitation, primarily potassium chloride (KCl), was noted. SS bands released an order of magnitude more ions than other components, which was attributed to the welding process used during manufacture. Furthermore, surface roughness did not correlate with ion release.

Utilizing artificial saliva as well, Ganidis et al. [44] studied stainless steel (SS), nickel-titanium (NiTi), and copper-nickel titanium (CuNiTi) dental archwires, which were immersed in artificial saliva. After immersion, the liquid was analyzed revealing that the leachates were mainly enriched with chromium (Cr) and nickel (Ni) ions after 30 days of aging, as the pH decreased. At a pH of 3.5, the highest level of ion release was observed. However, regardless of the material type or aging conditions, the levels of released ions never exceeded the average daily dietary intake.

Laird et al. [45] examined five archwires in different buffer solutions, each with a different pH level. The analyzed results showed that the average nickel release increased with time and decreased with pH, with coated archwires showing overall less metal ion increase than their uncoated counterparts.

Similarly, Osmani et al. [46] examined the effect of different pH levels on six types of archwires: nickel-titanium (NiTi), coated NiTi, stainless steel (SS), nickel-free SS, cobalt-chromium (CoCr), and titanium-molybdenum (TMA). The wires were immersed in artificial saliva. After each period, the release of metal ions was measured. Results showed that NiTi released more titanium (Ti) and nickel (Ni) ions compared to coated NiTi. SS released more iron (Fe), chromium (Cr), and Ni compared to nickel-free SS. CoCr released a high concentration of cobalt (Co) and lower amounts of Cr, Ni, and molybdenum (Mo) compared to the Mo and Ti released by TMA. Overall, metal release from dental orthodontic alloys in vitro was lower at pH 6.6 and in the hypoallergenic equivalents compared to standard dental alloys. Another evaluation of the effects of varying pH on ion release, this time from a nickel-chromium (NiCr) alloy, was conducted by Al-Jammal et al. [47]. They divided the alloy specimens into four groups according to the artificial saliva’s pH. Measurements were taken after immersion using atomic absorption spectroscopy (AAS). The results showed that the highest rate of Ni and Cr ion release occurred at pH 2.5 across all immersion times, with Ni being released in greater amounts than Cr. The authors concluded that metal release varies at different pH levels, with the highest release occurring at lower pH, and Ni release being greater than Cr release.

To compare metal ion emissions, Chikhale et al. [48] immersed archwires made of titanium-molybdenum (TMA) and nickel-titanium (NiTi) alloys in artificial saliva. Their findings indicated that nickel ion release from the NiTi wires were higher than from the TMA wires. However, the TMA wires released a higher amount of titanium ions than the NiTi wires. Despite the metal ion release, the authors found that the amounts did not exceed safety limits.

Another study that also included brackets was conducted by Aiswareya et al. [29]. In that study, nickel-titanium (NiTi) and stainless steel (SS) wires were attached to SS and ceramic brackets and immersed in artificial saliva. The release of nickel and chromium ions was quantified using a flame atomic absorption spectrometer (FAAS). A cytotoxicity assessment was also performed on a human cervical cancer cell line (HeLa). The wires attached to the SS brackets showed a significantly greater release of nickel and chromium ions. However, when comparing the wires themselves, the authors found no significant differences in ion release.

Understanding how variations in saliva pH influence ion release is also important, as investigated by Kao et al. [49]. They studied the cytotoxicity of fluoride corrosion extracts from SS and heat-activated NiTi archwires on human osteosarcoma cells (U2OS). They tested wires corroded in artificial saliva solutions at different pH levels. The authors concluded that fluoride-containing agents could pose cytotoxic risks for patients using these wires, emphasizing the need for caution. Similarly, Senkutvan et al. [33] evaluated nickel ion release from four types of archwires (NiTi, SS, CuNiTi, and ion-implanted NiTi) immersed in artificial saliva. Nickel release decreased over time and remained below levels that could trigger allergic reactions. They concluded that while these archwires release ions in acidic environments, they are safe for clinical use.

**Table 1:** Composition of artificial saliva used in the studies analyzed in this review.

Artificial Saliva Composition	References
PBS (phosphate buffer saline); 4.6 pH PBS + 0.001% NaF; 4.8 pH PBS + 0.01% NaF; 5 pH PBS + 0.1% NaF; 5.6 pH	[41]
0.844 mg Sodium chloride; 1.2 mg Potassium chloride; 0.146 mg Calcium chloride anhydrous; 0.052 mg Magnesium chloride 6 H_2_O; 0.34 mg Potassium phosphate dibasic; 60 mg 70% Sorbitol solution; 2 mg Methyl paraben; 3.5 mg Hydroxyethyl cellulose	[48] [30] [17]
Sodium chloride 0.4 g, potassium chloride 1.21 g, sodium hypo phosphate 0.78 g, sodium sulfide 0.005 g, urea-1 g, distilled water, and deionized water 1000 mL.	[33] [29]
Neutral solution: 1.5 mM Ca (Calcium), 0.9 mM P (Phosphorus), 20 mM Tris buffer, and 150 mM potassium chloride, pH 7.0 Acid solution: 2 mM Ca, 2 mM P, and 74 mM acetate buffer, pH 4.3	[32]
0.4 g KCl; 0.4 g NaCl; 0.906 g CaCl_2_·2H_2_O; 0.69 g NaH_2_PO_4_·2H_2_O; 0.005 g Na_2_S·9H_2_O; 1 g CO(NH_2_)_2_	[18]
Sodium chloride (0.84 mg/100 mL), Potassium chloride (1.2 mg/100 mL), Magnesium chloride (0.052 mg/100 mL), Calcium chloride (0.146 mg/100 mL), Potassium dihydrogen phosphate (0.34 mg/100 mL), Sorbitol solution (70%) at a volume of 60 mL, and Hydroxyethyl cellulose (3.5 mg/100 mL)	[49]
1.5 g/L KCl, 1.5 g/L NaHCO_3_, 0.5 g/L 0.5 g/L KSCN, and 0.9 g/L lactic acid	[37] [42] [46]
7.69 g of K_2_HPO_4_, 2.46 g of KH_2_PO_4_, 5.3 g of NaCl, and 9.3 g of KCl added to 1000 mL of distilled water	[47]

Sodium chloride (NaCl); potassium chloride (KCl); monosodium phosphate (NaH_2_PO_4_); water (H_2_O); sodium sulfide (Na_2_S); urea (CO(NH_2_)_2_); calcium chloride (CaCl_2_); sodium bicarbonate (NaHCO_3_); potassium thiocyanate (KSCN).

However, saliva in in vivo oral conditions is not static, which is an important factor to consider. Using a novel approach, Mikulewicz et al. [50] developed a thermostatic glass reactor with a constant artificial saliva flow rate to simulate the oral environment and measured nickel release from stainless steel (SS) archwires. Their findings indicated that the total nickel released was well below toxic levels, confirming the safety of SS wires.

Another important factor influencing ion release from nickel-containing archwires is the use of various oral hygiene products by patients. To account for this, Jamilian et al. [30] investigated nickel and chromium ion release from SS and round NiTi archwires immersed in three solutions (Oral B^®^, OrthoKin^®^, and artificial saliva). Their results showed a significant increase in ion release over time, with artificial saliva showing the lowest levels. They also noted that SS wires released ions at a slower rate than NiTi wires.

Similarly, Mirhashemi et al. [51] studied how several different types of mouthwashes would affect the release of metal ions from orthodontic archwires. They found that Listerine caused the highest release of ions, while Oral B^®^ showed the lowest amount of ion release.

Zubaidy and Hamdany [52] conducted a study to investigate how magnetically treated water (MTW) might affect the release of nickel ions from stainless steel (SS) archwires. The results showed that the MTW group released significantly fewer nickel ions from the SS orthodontic archwires compared to the mouthwash group. Thus, the authors concluded that MTW could be a safer alternative to commercially available mouthwashes during orthodontic treatment.

However, mouth hygiene products are not the only consumables that may influence ion release. In their study, Erwansyah et al. [53] demonstrated that snake fruit extract (*Salacca zalacca*) could inhibit nickel ion release from SS wires, particularly at a concentration of 300 ppm, suggesting a potential protective effect.

Table 2 provides a general overview of each in vitro study included in this work, including the material composition of the studied archwires, their brand, the released ions analyzed, exposure time, and the methods used to examine the archwires after immersion.

Considering that the shape of the archwire is also a decision that every orthodontist must make during treatment, this prompts the question of whether wire shape plays a role in ion release. In a study, Azizi et al. [17] compared ion release from round and rectangular nickel-titanium (NiTi) wires. Rectangular wires released significantly more ions, particularly during the first hour of immersion in artificial saliva. The authors concluded that wire shape influences ion release under simulated oral conditions.

Despite study results showing that the levels of ion release do not exceed safety limits, it is nevertheless important to conduct studies that examine whether released metal ions might be toxic when different tissue cells are directly exposed to them. Dugo et al. [54] conducted such a study in which they investigated the toxic effects of metal ions from NiTi and SS orthodontic appliances (NiTi archwires and SS bands, brackets, and ligatures) on four different types of cell lines (epithelial lingual—CAL 27, hepatic—HepG2, colon—CaCo-2, and stomach carcinoma—AGS). Regardless of exposure duration, the majority of eluates exhibited harmful effects on CAL 27 cells across the whole concentration range, although CaCo-2 showed the highest resistance. All examined samples produced free radicals in AGS and HepG2 cells, although the greatest concentration (2×) reduced the amount of free radicals produced in comparison to the lowest concentrations. A small pro-oxidant effect on DNA and slight genotoxicity were demonstrated by eluates containing Cr, Mn, and Al; nevertheless, these effects were not strong enough that the human body could not resist them. The impact of metal ions found in certain eluates on the toxicity measured is demonstrated by a statistical analysis of data on chemical composition, cytotoxicity, ROS, genotoxicity, and pro-oxidative DNA damage. While Mn and Cr have a significant impact on hydroxyl radicals, which not only produce ROS but also cause single strand breaks in supercoiled plasmid DNA, Fe and Ni are responsible for producing ROS. However, the cytotoxic effect of the examined eluates is caused by Fe, Cr, Mn, and Al.

One of the more recent studies, authored by Thiyagarajan et al. [55], utilized electrochemical techniques to analyze and evaluate the rate of nickel release from different types of orthodontic archwires (NiTi, SS, and CuNiTi). The archwires were immersed in artificial saliva for three days and then analyzed. Results confirmed that NiTi and CuNiTi wires exhibited greater corrosion resistance than SS wires. The authors concluded that saliva could affect the corrosion resistance of these types of wires. Nickel ion release was found to be negligible.

### 3.2. In Vivo Studies of Ni-Containing Archwires

The in vivo studies examined in this review focus on nickel-containing archwires that have been clinically used for various treatment periods (ranging from 7 days to 18 months). They are organized in this section according to the maximum duration of archwire use, concluding with two statistical studies on nickel release dynamics. Table 3 provides a general overview of each in vivo study included in this work—including the material composition of the archwires, their brand, the types of released ions studied, the duration of clinical use, and the methods employed to analyze the archwires post-application.

Orthodontic appliances, particularly those containing nickel, have been extensively investigated due to their potential to cause sensitization and release metal ions into the oral environment. Sensitization to allergens, such as nickel, has long been a well-documented concern in orthodontic practice. To understand the mechanisms behind nickel release, Ghazal et al. [56] analyzed the surface morphology and nickel ion release of superelastic and heat-activated NiTi wires. They found that both types of wires released comparable amounts of nickel ions after 30 days of clinical use, although superelastic wires exhibited greater surface roughness. Surface roughness increased with clinical use, but wires retrieved from both groups released fewer nickel ions after immersion in artificial saliva. This suggests that while nickel release occurs, it may diminish over time.

Ibañez et al. [57] investigated the temporal dynamics of metal ion release and its relationship with salivary pH in heat-activated NiTi archwires and stainless steel (SS) archwires. They found that the metal ion release peaked but remained within acceptable limits. Salivary pH decreased to an acidic level after three months of treatment but returned to an alkaline state after six months. This suggests that while orthodontic appliances can alter the oral environment, the body may adapt over time.

Almasry et al. [58] evaluated nickel ion release from round thermoactive NiTi archwires within the first two months of treatment. They observed a slight increase in nickel ion release but concluded that the levels did not exceed safety thresholds. This supports the idea that while nickel release occurs during orthodontic treatment, it generally remains within safe limits.

Bass et al. [59] investigated the relationship between orthodontic therapy using stainless steel (SS) and nickel-titanium (NiTi) archwires and nickel sensitization in 1993, focusing on patients with preexisting nickel sensitivity. Among 29 patients, 5 (all female) initially tested positive for nickel sensitivity, and 2 additional patients developed sensitivity during treatment. The study concluded that nickel sensitivity is more prevalent among females and that orthodontic appliances, while having minimal effects on overall oral health, may induce sensitivity in certain cases.

Lages et al. [60] expanded the investigation of nickel release by measuring salivary metal levels, including nickel, in patients with metal and esthetic orthodontic appliances (SS brackets and heat-activated NiTi archwires). In their retrospective cohort study, they found no significant differences in nickel levels between the control group and those with metal appliances, or between the esthetic and control groups. However, the type of appliance significantly influenced nickel concentration, emphasizing the importance of material choice in metal ion release.

Amini et al. [61] conducted a hypothesis test to determine whether salivary metal ion content differs between subjects with fixed orthodontic appliances and their same-gender siblings who were not undergoing orthodontic treatment. The subjects were treated with nickel-titanium (NiTi) and stainless steel (SS) archwires, while the remaining components (bands and brackets) were made of SS. A saliva sample was collected from each subject, with the sibling’s saliva serving as the control. The authors found that nickel (Ni) content was significantly higher in the study group compared to the control group, while chromium (Cr) levels did not show a statistically significant difference. Considering the limitations of an in vivo study, the authors concluded that the presence of fixed orthodontic appliances leads to an increase in metal ion concentrations in saliva.

**Table 2:** General overview of the nickel-containing archwires and methods used in the reviewed in vitro studies.

Material	Brand and Manufacturer	Releasedions Studied	Study Media	Exposure Time	Method of Analysis	Reference
NiTi	Nitinol N Memory-Metalle 0.5 × 0.5 mm (GmbH, Weil am Rhein, Germany) Nitinol S Memory-Metalle foil 0.05 and 1 mm (GmbH, Weil am Rhein, Germany) Sentalloy standard 0.46 × 0.46 mm (GAC International Inc., Bohemia, NY, USA) Neo sentalloy standard 0.46 × 0.63 mm (GAC International Inc., Bohemia, NY, USA)	Ni	Artificial saliva (fluoridated and non-fluoridated)	7 d	Thin layer activation X-ray photoelectron spectroscopy	[41]
NiTi	Round and rectangular NiTi archwires 0.020 in round and 0.016 × 0.016 in rectangular (Ortho Technology, Tampa, FL, USA)	Ni, Ti	Artificial saliva	1 h, 24 h, 7 d, 21 d	Inductively coupled plasma atomic emission spectrometry	[17]
NiTi, TiMo	17 × 25 in NiTi archwire a 17 × 25 inTMA archwire (Modern Orthodontics, Ludhiana, India)	Ni, Ti	Artificial saliva	90 d	Atomic absorption spectrometry	[48]
SS, NiTi, TiMo	SS (American Orthodontics, Sheboygan, WI, USA) NiTi (Neo Sentalloy, GAC, West Columbia, USA) TiMo (Beta Blue, Highland Metals, Bangkok, Thailand)	Ni, Ti	Mouthwashes (brands not specified)	1 d, 4 d, 7 d, 14 d	Inductively coupled plasma mass spectrometry Scanning electron microscopy	[43]
NiTi, CuNiTi	Ni titanium (Ti) Memory Wire 0.016 in (American Orthodontics) Damon Optimal-Force Cu Ni-Ti 0.016 in (Ormco) Tanzo Cu NiTi 0.016 in (American Orthodontics) Flexy NiTi Cu 0.016 in (Orthometric)	Ni, Cu	Neutral and acid solution	7 d	Graphite furnace atomic absorption spectrometry Inductively coupled plasma atomic emission spectrometry	[32]
NiTi, coated NiTi, SS, Ni-free SS, CoCr, TMA	BioForce Sentalloy (Dentsply GAC, New York, NY, USA) High Aesthetic (Dentsply GAC, New York, NY, USA) Remanium (Dentaurum, Ispringen, Germany) Noninium (Dentaurum, Ispringen, Germany) Elgiloy (Dentaurum, Ispringen, Germany) Rematitan Special (Dentaurum, Ispringen, Germany)	Ni, Ti	Artificial saliva	3 d, 7 d, 14 d, 28 d	Inductively coupled plasma mass spectrometry	[46]
NiTi, CuNiTi, SS	N/A	Ni	Artificial saliva	3 d	Cyclic voltammetry electrochemical impedance spectroscopy polarization (Tafel) plot	[55]
NiTi, Esthetic wires, SS	0.019 × 0.025 in NiTi (Ormco, Glendora, CA, USA) 0.019 × 0.025 in FLI wire (Rocky Mountain Orthodontics Denver, CO, USA) 0.019 × 0.025 in Iconix (American Orthodontics Sheboygan, WI, USA) 0.019 × 0.025 in Bio-Active RC (GC Orthodontics TOMY Inc., Fuchu City, Tokyo) 0.019 × 0.025 in SS (3 M Unitek, St. Paul, MN, USA)	Ni, Cr	Buffer solutions with varying pH (4, 5.5, and 7)	4 wks, 13 wks	Inductively coupled plasma mass spectrometry	[45]
NiTi, SS	Rematitan^®^ LITE ideal arches 0.43 × 0.64 mm (Dentaurum, PA, USA)	Fe, Ni, Cr, Mn, Al, Ti, Cu	Artificial saliva	3 d, 7 d, 14 d	Scanning electron microscopy with energy dispersive spectroscopy Inductively coupled plasma mass spectrometry	[42]
NiTi, SS	Wire SS Upper 016 Form III 0.016 × 0.016 Wire NiTi Form I Upper 016 0.016 × 0.016 Tanzo^®^ Copper Nickel Titanium (Tanzo Low Wire Upper 016) 0.016 × 0.016 Tru-Arch^®^ UM 0.016 × 0.016 (Ormco) Tru-Arch^®^ CuNiTi 35 °C UL 0.016 × 0.022 (Ormco)	Ni, Mn, Cr, Mo, Ti	Artificial saliva	7 d, 30 d	Inductively coupled plasma optical emission spectrometer	[44]
NiTi, SS	SS (Fe-18Cr-8Ni) 0.010/0.014/0.016 × 0.022 in (3M Unitek, Monrovia, Calif) Heated activated Nitinol 0.016/ 0.016 × 0.022 in (3M Unitek, Monrovia, Calif)	Ni, Ti, Cr	Artificial saliva	1 h, 24 h	Atomic absorption method	[49]
NiTi, SS	NiTi 0.016 × 0.022 in (American orthodontics, Sheboygan, WI, USA) Stainless steel 0.016 × 0.022 in (American orthodontics, Sheboygan, WI, USA) Ion implanted NiTi 0.016 × 0.022 in (GAC international, Bohemia, NY, USA) Copper NiTi 0.016 × 0.022 in (Ormco)	Ni	Artificial saliva	7 d, 14 d, 21 d	Atomic absorption method	[33]
NiTi, SS	SS rectangular archwires 0.017 × 0.025 in (Ormco) NiTi rectangular archwires 0.017 × 0.025 in (Ormco)	Ni, Cr	Artificial saliva	7 d, 14 d, 1 mo	Flame atomic absorption spectrometry	[29]
NiTi, SS	Nitinol 0.4 mm (Dentaurum, Germany) SS304 0.4 mm (Tiger Ortho, Boston, MA, USA)	Ni, Ti, Cr, Mo, Mn	Fusayama–Meyer solution	N/A	Potentiodynamic and potentiostatic polarizations Energy dispersive X-ray atomic adsorption spectroscopy	[18]
NiTi, SS	SS 0.018 in diameter (American Orthodontics, Sheboygan, WI, USA) NiTi 0.018 in diameter (American Orthodontics, Sheboygan, WI, USA)	Ni, Cr	Oral B^®^, Orthokin^®^, Artificial saliva (SaliLube^®^, Sinphar Pharmaceutical Co., Ltd., Taipei, Taiwan)	1 h, 6 h, 24 h, 7 d	Atomic absorption method	[30]
NiTi, SS	N/A	Ni, Cr	Oral B^®^, Oral B^®^ 3D White Luxe, Listerine, Listerine Advanced White	1 h, 6 h, 24 h, 168 h	Atomic absorption spectroscopy	[51]
SS	N/A	Ni, Cr	Snakefruit extract (*Salacca zalacca*)	24 h	Atomic absorption spectrophotometry	[50]
SS	SS archwires 0.016 × 0.022 in (Dentarum, Germany)	Ni	Magnetically treated water, OrthoKin^®^	24 h, 2 wks, 4 wks	Scanning electron microscopy Atomic absorption spectrometry	[52]
NiCr (alloy)	N/A	Ni, Cr	Artificial saliva	12 d, 24 d, 36 d	Atomic absorption spectroscopy	[47]

Nickel-Titanium (NiTi); nickel-titanium with coating (coated NiTi); copper nickel-titanium (CuNiTi); stainless steel (SS); nickel-chromium (NiCr); nickel-free stainless steel (Ni-free SS); titan-molybdenum (TiMo); cobalt-chromium (CoCr); titanium-molybdenum alloy (TMA); days (d); inches (in).

**Table 3:** General overview of the nickel-containing archwires and analytical methods used in the reviewed in vivo studies.

Material	Brand and Manufacturer	Released Ions Studied	Study Media	Exposure Time	Method of Analysis	References
NiTi	NiTi Force I^®^ 0.019 × 0.025 in (American Orthodontics, Sheboygan, WI, USA) Therma-Ti Lite^®^ 0.019 × 0.025 in (American Orthodontics, Sheboygan, WI, USA)	Ni	Oral environment	1 mo	Scanning electron microscopy Atomic force microscopy Atomic absorption spectrophotometry	[56]
NiTi, CuNiTi	Superelastic (austenitic) NiTi 0.016 × 0.022 in Heat-activated NiTi 0.016 × 0.022 in Heat-activated CuNiTi 0.016 × 0.022 in	Ni	Oral environment	6 wks, 8 wks	Energy dispersive X-ray Dynamic modeling	[62]
NiTi, Rh-coated NiTi, SS	Heat-activated nitinol archwire (Abzil, São José do Rio Preto, SP, Brazil) Heat-activated nitinol archwire coated with rhodium polymer 0.014 in (BioActive, Crystal 3D, São Carlos, SP, Brazil)	Ni, Cr, Fe, Cu	Oral environment	1–6 mo	Total reflection X-Ray fluorescence technique	[60]
NiTi, SS	N/A	N/A	Oral environment	3 mo	Nickel patch Gingival index Plaque index Intraoral photographs	[59]
NiTi, SS	Ni–Ti heat-activated wires 0.016 in (3 M^™^ Unitek^™^ mark) Stainless steel wires 0.016 × 0.022 in (3 M^™^ Unitek^™^ mark)	Ni, Ti	Oral environment	1 mo	Coupled plasma optical emission spectroscopy Scanning electronic microscopy	[57]
NiTi, SS	Round thermoactive archwires 0.016 in (Equire Thermo-Aktive, Dentaurum, Germany)	Ni	Oral environment	7 d, 1 mo, 2 mo	Atomic absorption spectrophotometry	[58]
NiTi, SS	Stainless steel CrNi Superelastic (austenitic) NiTi Thermodynamic heat-activated NiTi Thermodynamic heat-activated CuNiTi TriTanium^™^ Bio-active^™^	Ni	Oral environment	6 wks, 8 wks	Scanning electron microscopy with energy dispersive spectroscopy Dynamic modeling	[63]
NiTi, SS	Pre-adjusted roth stainless steel brackets 0.018 in (Discovery, Dentaurum, Pforzheim, Germany) Stainless steel orthodontic bands (Unitek/3M, Monrovia, CA, USA) Nitinol (Ormco Corporation, Orange, CA, USA) Stainless steel archwires (Remantium; Dentaurum)	Ni, Cr	Oral environment	12–18 mo	Atomic absorption spectrophotometry	[61]

Nickel-Titanium (NiTi); nickel-titanium with coating (coated NiTi); copper nickel-titanium (CuNiTi); stainless steel (SS); nickel-chromium (NiCr).

Based on the statistical analysis of nickel dynamics in various nickel-containing orthodontic archwires following clinical application in the intraoral environment, studies were conducted to evaluate how nickel content changes with clinical use and to provide practical recommendations regarding the duration of use for the studied types of archwires [62,63].

The 2019 study [62] conducted a statistical analysis of austenitic NiTi, heat-activated NiTi, and heat-activated CuNiTi types of orthodontic wires. The wires were categorized into four groups: as-received autoclaved (S0), as-received (S1), intraorally used for up to six weeks (S2), and intraorally used for over eight weeks (S3). The nickel content was assessed by analyzing multiple visually distinct areas along the length of the wires. To quantify the nickel content for statistical analysis, the study utilized both global (average over all samples) and local (focused on the most corroded areas) surface. The global measurements showed no significant differences between groups S0 and S1, nor between S1 and S2/S3. However, the local measurements revealed statistically significant changes between groups S1, S2, and S3. Based on the local measurements, a model of nickel content dynamic behavior was constructed. However, the authors noted that this model only provides a general estimation, and that practicing orthodontists should consider the patient’s specific health circumstances when applying these findings.

Building on this, the 2025 study [63] examined the same types of wires, with the addition of stainless steel (SS) wires and multi-force wires. The archwires were categorized into three groups: as-received, clinically used for up to six weeks, and clinically used for over eight weeks. The findings highlighted that each alloy exhibits unique nickel release patterns, influenced by both material composition and environmental conditions. The study showed that, due to their stability, SS-CrNi, heat-activated NiTi with Cu (HA-NiTi-Cu), and TriTanium^™^ are suitable for long-term use. In contrast, due to higher nickel release, superelastic NiTi, heat-activated NiTi without Cu (HA-NiTi), and Bio-Active^™^ are better suited for short- to medium-term use. However, the authors emphasized that this model provides only general recommendations, and a patient’s individual condition should always be considered.

These studies collectively demonstrate that orthodontic appliances, particularly those containing nickel, can release metal ions and potentially induce sensitization, especially among females. However, the levels of nickel released are generally within safe thresholds, and factors such as surface roughness, appliance type, and treatment duration influence the extent of release. While nickel sensitivity remains a concern, appropriate material selection and regular monitoring can mitigate risks, ensuring that orthodontic treatment is safe for most patients.

Overall, factors such as fluoride exposure, pH levels, immersion time, saliva dynamics, oral hygiene products, dietary elements, and wire geometry significantly impact nickel ion release. These variables influence clinical safety and inform material selection in orthodontics

## 4. Insights from the Literature

The 24 reviewed studies, primarily from recent years, collectively provide a comprehensive understanding of nickel release from nickel-containing orthodontic appliances, particularly nickel-titanium (NiTi) archwires, with and without copper, and stainless steel (SS) archwires. These are the most commonly used in fixed orthodontic treatment techniques and have significant implications for biocompatibility and patient safety. Over the years, research has evolved from initial concerns about the biocompatibility of NiTi alloys to a more nuanced understanding of the factors influencing nickel ion release in both in vitro and in vivo environments.

### 4.1. Biocompatibility and Short-Term Safety of NiTi Alloys

The study by Wever et al. [22] was pivotal in establishing the short-term biocompatibility of nickel-titanium (NiTi) alloys, which are commonly used to manufacture orthodontic appliances, such as archwires. Their comprehensive in vitro and in vivo tests demonstrated that NiTi alloys exhibit low cytotoxicity, minimal sensitization potential, and high corrosion resistance, making them suitable for clinical use. These findings are supported by Kovac et al. [64], who confirmed that ion release from NiTi archwires and stainless steel (SS) brackets remains below recommended daily intake thresholds, even after prolonged exposure. However, the observation that ion concentrations were higher in debris than in artificial saliva underscores the importance of considering localized effects, such as the accumulation of food debris infused with nickel ions, which may contribute to hypersensitivity reactions in susceptible individuals. Metal debris is also an important factor to consider regarding biocompatibility, as outlined in a review by Matusiewicz [65]. The studies they examined show that corrosion occurs when archwires are exposed to the intraoral environment (simulated or real), leading to the release of metallic debris, which can accumulate over time, especially in patients with poor oral hygiene.

The in vitro studies reviewed here generally demonstrate that the amount of metal ions released, including nickel, does not reach levels considered dangerous. However, as these studies are conducted in vitro, they cannot fully replicate the unique oral environment of each patient and should therefore be regarded as general indicators.

This is where in vivo studies are essential, as they first confirm that nickel-containing orthodontic archwires do release metal ions in generally safe amounts, but they also reveal the potential to induce sensitization, with a higher incidence observed in females than in males. While nickel sensitivity remains a concern, appropriate material selection and regular monitoring can mitigate risks, ensuring that orthodontic treatment is safe for most patients.

### 4.2. Influence of Fluorides, pH, and Saliva Dynamics

Fluoride exposure has emerged as a significant factor influencing nickel ion release. Cioffi et al. [40] and Mirjalili et al. [18] demonstrated that, while NiTi archwires resist phase transformation under tensile stress, prolonged exposure to fluoridated media significantly increases ion release. This finding is clinically relevant, as fluoride-containing oral hygiene products are commonly used by orthodontic patients. Similarly, Kao et al. [49] highlighted the cytotoxic risks associated with fluoride corrosion extracts at low pH levels, emphasizing the need for caution when using acidic fluoride agents in patients with NiTi archwires. The risk posed by mouthwashes in increasing ion release can be mitigated by using magnetically treated water, as evidenced by the study from Zubaidy and Hamdany [52].

**Figure 2:**
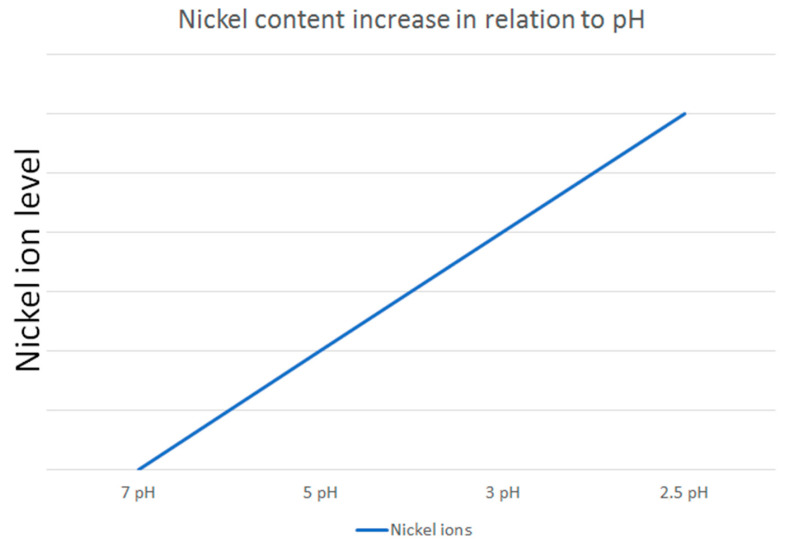
An illustrative example of how nickel ion release varies in relation to pH levels, based on the reviewed studies.

The dynamic nature of saliva in the oral environment further complicates the assessment of nickel release. Mikulewicz et al. [50] developed an innovative thermostatic reactor to simulate oral conditions, confirming that nickel release from stainless steel (SS) archwires remains below toxic levels. This study highlights the importance of replicating in vivo conditions in in vitro experiments to obtain clinically relevant data. Additionally, Senkutvan et al. [33] and Ibañez et al. [57] demonstrated that while nickel release increases in acidic environments, it decreases over time and remains within safe limits, suggesting that the oral environment may adapt to the presence of orthodontic appliances. The study by Osmani et al. [46] supports this finding by demonstrating that metal ion release is generally lower at higher pH levels, further confirming that acidic conditions promote greater ion release (Figure 2).

### 4.3. Role of Surface Morphology, Wire Shape, and Material Selection

Surface morphology and wire shape were identified as critical factors influencing nickel release. Didovic et al. [42] and Aiswareya et al. [29] found that surface roughness and production techniques significantly affect ion release, with stainless steel (SS) bands exhibiting higher ion release compared to their nickel-titanium (NiTi) counterparts, primarily due to welding processes. Azizi et al. [17] further demonstrated that rectangular NiTi wires release more ions than round wires, highlighting the need for orthodontists to consider wire shape when selecting materials for patients with nickel sensitivity.

The choice of orthodontic materials also plays a crucial role in minimizing nickel release. Lages et al. [60] found no significant differences in nickel levels between patients with metal and esthetic fixed orthodontic appliances, suggesting that esthetic alternatives may be a viable option for nickel-sensitive patients. However, the study by Bass et al. [59] highlighted that nickel sensitivity is more prevalent in females and may be exacerbated by orthodontic treatment, emphasizing the need for careful patient monitoring and material selection.

### 4.4. Clinical Implications and Future Directions

The development of predictive models, as proposed in studies [62,63], could provide orthodontists with valuable tools to assess nickel release dynamics and tailor treatment plans based on individual patient sensitivity. Based on these studies and the broader body of reviewed research, the following clinical recommendations can be made for each type of archwire:

SS CrNi (stainless steel chromium–nickel): Suitable for long-term applications, as it maintains stable nickel release after the initial period. It can be used for treatments that extend over several months.

NiTi Superelastic: Best suited for applications up to 4–6 weeks, as the peak nickel release during the early stages of treatment may support consistent force application. However, it may not be ideal for long-term use due to potential sensitization risks.

Heat-Activated NiTi (without copper): Appropriate for applications up to 6–8 weeks, given the high initial release. After this period, replacement is advisable if nickel release stability is a concern.

Heat-Activated NiTi (with copper): suitable for long-term treatments (over several months), as it exhibits a steady release profile that eventually plateaus, reducing the risk of excessive nickel exposure.

TriTanium^™^: recommended for long-term use, as the nickel content stabilizes over time, making it ideal for treatments extending over several months.

Bio-Active^™^: Effective for short- to medium-term treatments (up to 4–6 weeks), as the initial high release can support early treatment forces. However, it may require replacement if long-term stability is necessary.

From the reviewed studies, it is clear that nickel ion release is significantly influenced by variables such as fluoride exposure, pH levels, immersion duration, saliva dynamics, oral hygiene products, dietary components, and wire design. These factors affect both clinical safety and the selection of orthodontic materials, emphasizing the need for careful assessment in clinical practice.

Future research should focus on long-term in vivo studies to better understand the cumulative effects of nickel exposure over the course of orthodontic treatment. Additionally, the development of novel materials with enhanced corrosion resistance and reduced ion release potential could further improve patient safety. Investigations into the protective effects of natural compounds, such as snake fruit extract [53], and the optimization of surface treatments, such as pre-passivation [18], also hold promise for mitigating nickel release.

## 5. Concluding Remarks

The current review is by nature of its design limited and does not claim to be universally applicable. Therefore, one must consider the relative strength of the evidence.

The reviewed studies consistently agree that nickel-containing archwires release nickel ions in both in vivo and in vitro environments. Although the daily amounts remain below toxic thresholds, posing no immediate health risks, the potential for nickel sensitization and allergy development in previously unaffected individuals warrants caution. The selection of archwires should consider factors such as pH levels, saliva dynamics, wire shape, oral hygiene practices, patient allergies, and dietary habits. While short-term use of these archwires is generally considered safe, continuous patient monitoring is essential to detect potential sensitization. Although short-term nickel release from stainless steel (SS) and nickel-titanium (NiTi) wires is well-documented, long-term exposure studies, particularly those combining in vitro and in vivo environments, remain limited. Further research is necessary to assess the prolonged effects of nickel release and to enhance clinical safety guidelines.

To establish causal relationships in metal ion release, in vivo monitoring of ions such as nickel (Ni) and chromium (Cr) is critical. Analysis must extend beyond total elemental quantification to include the chemical form, oxidation state, and organometallic properties of the ions. Advancements in trace element analytical techniques are imperative, enabling species-specific separation and detection at subnanogram to picogram environmental levels.

Furthermore, collaborative efforts among practitioners, researchers, and regulatory bodies are essential for developing evidence-based guidelines for orthodontic material selection. These efforts should prioritize patient safety and address the risks associated with metal ion release.

## Data Availability

Data are contained within the article.
